# *Terminalia bellirica* Fruit Extract Alleviates DSS-Induced Ulcerative Colitis by Regulating Gut Microbiota, Inflammatory Mediators, and Cytokines

**DOI:** 10.3390/molecules28155783

**Published:** 2023-07-31

**Authors:** Yao-Yao Li, Yu Cui, Wan-Rong Dong, Tian-Tian Liu, Gao Zhou, Yu-Xin Chen

**Affiliations:** Key Laboratory of Fermentation Engineering (Ministry of Education), Cooperative Innovation Center of Industrial Fermentation (Ministry of Education & Hubei Province), School of Biological Engineering and Food, Hubei University of Technology, Wuhan 430068, China

**Keywords:** *Terminalia bellirica*, ulcerative colitis, inflammation, IL-6/JAK2/STAT3

## Abstract

Ulcerative colitis (UC) is a chronic inflammatory disease significantly impacting patients’ lives. This study aimed to elucidate the alleviating effect of ethyl acetate extract (TBEA) from *Terminalia bellirica* fruit on UC and to explore its mechanism. TBEA was the fraction with the best anti-inflammatory activity screened using in vitro anti-inflammatory assays, and HPLC initially characterized its composition. The mice model of ulcerative colitis was established after free drinking of 2.5% dextran sulfate sodium for six days, and the experimental group was treated with 50 mg/kg and 100 mg/kg TBEA for seven days. We found that TBEA significantly alleviated symptoms in UC mice, including a physiologically significant reduction in disease activity index and pathological damage to colonic tissue. TBEA dramatically slowed down oxidative stress and inflammatory process in UC mice, as evidenced by decreasing myeloperoxidase and malondialdehyde activities and increasing glutathione and catalase levels by reducing the concentrations of IL-6, IL-1β, TNF-α, and NO in UC mice, as well as by regulating key proteins in the IL-6/JAK2/STAT3 pathway. Meanwhile, TBEA maintained intestinal homeostasis by regulating intestinal flora structure. Our study provides new ideas for developing TBEA into a new drug to treat UC.

## 1. Introduction

UC is a typical inflammatory bowel disease (IBD), characterized by persistent inflammatory lesions in the mucosa and submucosa of the colon and rectum, which is persistent and prone to recurrent attacks [1]. The main clinical features of UC are diarrhea with blood and mucus, abdominal pain of varying degrees caused by rectal contraction, and even systemic toxicity in severe cases [2]. Most studies have shown that the host immune system, genetics, gut microbiome, and environment all play important roles in the pathogenesis of UC. Damage to the intestinal mucosal barrier disrupts the balance of four interrelated biological, mechanical, chemical, and immune barriers. Induced by such imbalance, the body produces severe chronic inflammatory response and even increases the risk of colon cancer [3]. Glucocorticoids, 5-aminosalicylic acid (5-ASA), and immunosuppressants are commonly used clinically to control UC inflammation and relieve symptoms. However, relapse after drug withdrawal, long-term efficacy decline, adverse reactions, and other problems related to these drugs remain to be solved [4]. A lack of long-term effective therapeutic drugs leads to some patients undergoing resection [5]. Therefore, the development of mild and effective treatment drugs is crucial.

Traditional Chinese medicine (TCM) is a kind of traditional medicine system with a long history and wide application. It is based on unique Chinese cultural theories and powerful practices and is characterized by multi-targeting and low side effects, which gives it a great advantage in the treatment of many chronic diseases [6]. Studies have shown that TCM plays an active role in improving intestinal flora, repairing the intestinal barrier, and maintaining intestinal health [7]. Recently, the potential efficacy and mechanisms of action of many TCM approaches for UC have been confirmed using scientific experiments. Zhang et al. demonstrated that Bilobalide from *Ginkgo biloba* could alleviate the severity of UC by improving physiological indices and pathological damage in UC mice, reducing inflammatory factor levels by inhibiting MAPK and Akt/NF-κB pathway activation and enhancing the intestinal barrier, as well as regulating intestinal flora [8]. A review summarized the most commonly used Chinese decoctions and medicines for the treatment of UC as *Bai-Tou-Weng-Tang, Shen-Ling-Bai-Zhu-San*, *Coptis chinensis* Franch., *Atractylodes macrocephala* Koidz., Fisch. and *Astragalus membranaceus* (Fisch.) Bge [9].

*Terminalia bellirica* fruit (TBF), a member of the *Combretaceae* family, is widely used in the Tibetan system of medicine and occupies an important position in TCM. The TCM system describes TBF as playing a role in clearing heat, detoxifying the blood as an astringent, nourishing the blood, and improving diarrhea and dysentery [10]. Modern studies suggest that it mainly contains tannins, flavonoids, terpenoids, steroids, and lignans and has pharmacological effects such as antioxidant, antidiabetic, anti-inflammatory, antibacterial, and anticancer [11,12]. The current therapeutic approaches for UC, reported as being available, mainly target features of inflammation, oxidative stress, intestinal mucosal barrier damage, and intestinal flora disorders [13]. It has been shown that TBF extract can inhibit LPS-induced inflammatory response in macrophages by affecting the MAPK/NF-κB pathway [14]. Therefore, research on the use of TBF to alleviate UC has a broad prospect. In addition, more in-depth in vivo studies on the anti-inflammatory effect of TBF are worth conducting.

This study used LPS-stimulated macrophages to select TBF extracts with optimal anti-inflammatory effects. Importantly, 5-ASA can alleviate mild to moderate ulcerative colitis and is a well-established drug for clinical utilization. Therefore, in this study, we used dextrose sodium sulfate (DSS)-induced ulcerative colitis mouse model as the research object and 5-ASA as the positive control drug to explore the alleviating effect of TBEA on inflammation and oxidative stress in UC mice. We compared the therapeutic effects with 5-ASA as well as the underlying molecular mechanisms, including the signal pathways involved and gut microbiota regulation.

## 2. Results

### 2.1. HPLC Chromatogram

**Figure 1:**
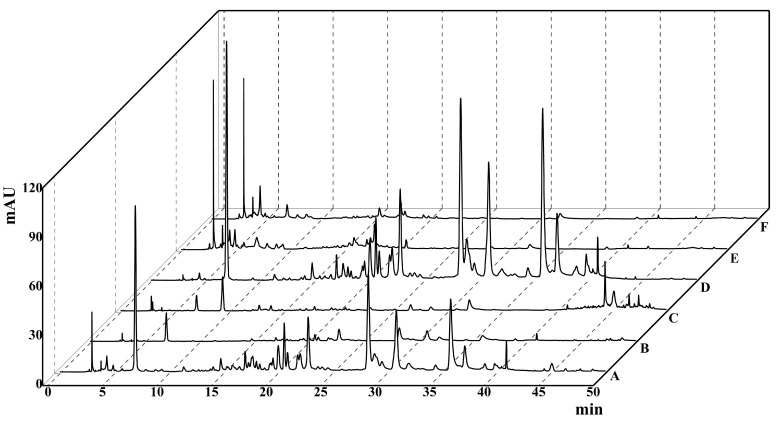
The HPLC chromatograms of TBFE and five extract fractions of *Terminalia bellirica*: A, 95% ethanol extract (TBFE); B, petroleum ether extract fraction (TBPE); C, dichloromethane extract fraction (TBFD); D, ethyl acetate extract fraction (TBEA); E, *n*-butanol extract fraction (TBNB); and F, aqueous phase extract fraction (TBFW).

**Figure 2:**
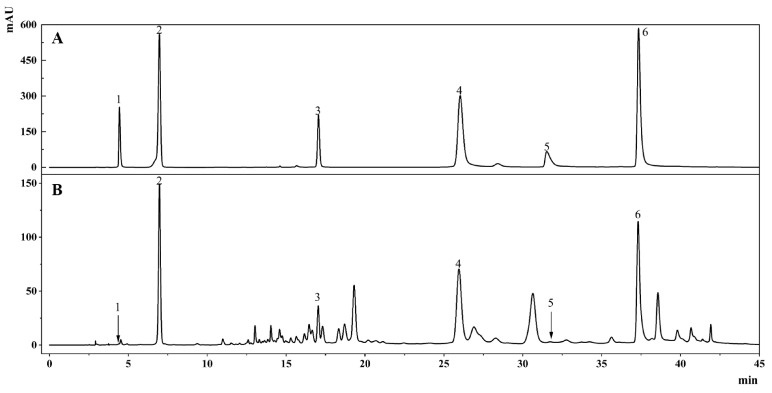
(**A**) HPLC chromatogram of the mixed standards. (**B**) HPLC chromatogram of the TBEA sample. (1: chebulic acid; 2: gallic acid; 3: corilagin; 4: chebulagic acid; 5: ellagic acid; and 6: chebulinic acid).

A comparison of the HPLC results for TBF 95% ethanol extract (TBFE) and five extract fractions of TBF showed that TBFE and TBEA were richer in various active substances than the other four extracted fractions (Figure 1), which may contribute to higher pharmacological activities. Comparing the peak retention times for the experimental sample with those for the mixed standards, it was tentatively determined that the extracted TBEA mainly contained gallic acid, corilagin, chebulagic acid, and chebulinic acid (Figure 2), confirming that the TBEA used in the experiments contains these active ingredients of TBF.

### 2.2. Effect of TBFE and Five Extract Fractions on Macrophage Viability and In Vitro Anti-Inflammatory Activity

**Figure 3:**
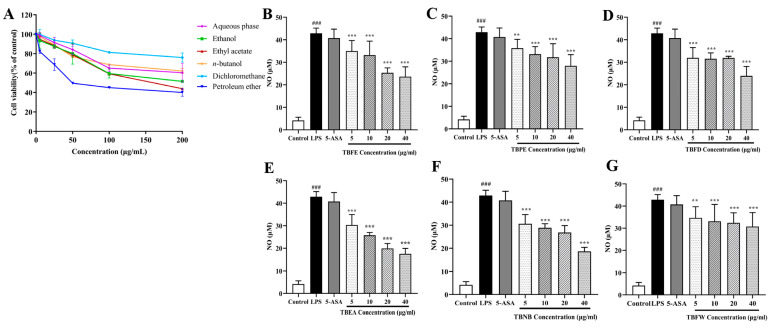
Effect of TBFE and five extract fractions on macrophage viability and in vitro anti-inflammatory activity. (**A**) Effect of six extracts on cell viability. (**B**) Effect of TBFE on LPS-induced NO content production by RAW264.7. (**C**) Effect of petroleum ether extract fraction (TBPE) on LPS-induced NO content. (**D**) Effect of dichloromethane extract fraction (TBFD) on LPS-induced NO content. (**E**) Effect of ethyl acetate extract fraction (TBEA) on LPS-induced NO content. (**F**) Effect of *n*-butanol extract fraction (TBNB) on LPS-induced NO content. (**G**) Effect of aqueous phase extract fraction (TBFW) on LPS-induced NO content. ^###^ *p* < 0.001 compared with the control group, and ** *p* < 0.01, *** *p* < 0.001 compared with the LPS group. Note: 5-ASA, 5-aminosalicylic acid. Data represent mean ± SD, *n* =3.

The MTT method was used to assess whether the extracts had an effect on the viability of macrophage RAW264.7. Figure 3A shows that, except for the petroleum ether extract, the extracts were less toxic to cells at concentrations below 50 μg/mL. The calculated IC_50_ values for each extraction fraction were obtained as TBFE, 198.151 μg/mL; TBPE, 74.847 μg/mL; TBFD, 568.706 μg/mL; TBEA, 158.599 μg/mL; TBNB,285.772 μg/mL; and TBFW, 205.324 μg/mL. Based on this result, safe concentrations were selected, and the experimental samples were screened using an in vitro anti-inflammatory activity assay during the experiments. The amount of NO produced by the cells after LPS stimulation was significantly increased in all treatment groups (*p* < 0.001) (Figure 3). The IC_50_ value of each extract fraction for inhibition of NO production was obtained as TBEA, 18.941 μg/mL; TBNB, 28.029 μg/mL; TBFE, 38.304 μg/mL; TBFD, 54.508 μg/mL; TBAP, 63.372 μg/mL; and TBPE, 75.930 μg/mL. Figure 3B–G shows that the reduction in NO production after inducing an inflammatory response in the cells was better in the TBEA-treated group than in the other treated groups, indicating that TBEA had the best in vitro anti-inflammatory activity among these extract fractions.

### 2.3. Amelioration of Physiological Characteristics and Colonic Injury in UC Mice with TBEA

**Figure 4:**
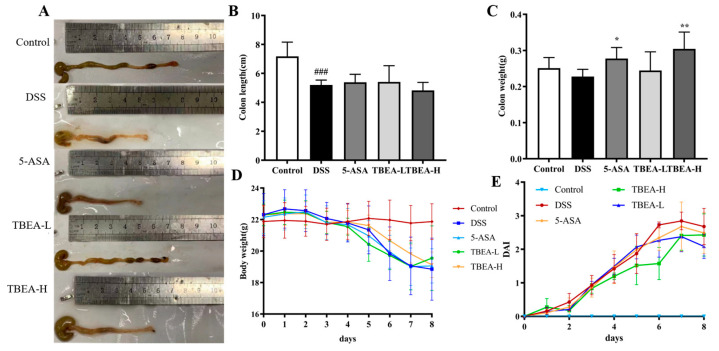
Changes in physiological characteristics of experimental mice from the five groups. (**A**) Macroscopic pictures of colons. (**B**) Colon length. (**C**) Colon weight. (**D**) Changes in body weight of mice. (**E**) Changes in DAI. TBEA-L, TBEA-low dose (50 mg/kg); TBEA-H, TBEA-high dose (100 mg/kg); 5-ASA, 5-aminosalicylic acid (200 mg/kg). ^###^ *p* < 0.001 compared with the control group, and * *p* < 0.05, ** *p* < 0.01 compared with the DSS group (*n* = 8).

In this study, TBEA, the component with the best anti-inflammatory activity in vitro, was selected as the best extract type for in vivo anti-ulcerative colitis activity, in which TBEA low-dose (TBEA-L, 50 mg/kg) and TBEA high-dose (TBEA-H, 100 mg/kg) were established. UC causes significant lesions in the colon, including congestion, edema, easy bleeding, and erosion, as can be observed in Figure 4A. Some swelling and congestion were observed in the colon tissue of the DSS group but not in the other groups, revealing that TBEA can prevent colonic injury. According to Figure 4B, the DSS group showed a significant shortening in colon length (*p* < 0.001). There was no statistically significant effect on colon length after TBEA treatment. As shown in Figure 4C, mice in the 5-ASA and TBEA-H groups showed significant improvement in colon weight compared with the DSS group (*p* < 0.05, *p* < 0.01). Still, the TBEA-L treatment did not show any effective improvement in colon weight, which is inferior to the TBEA-H treatment. Starting from the fourth day of the experiment, the body weight of mice in the DSS group began to decrease. At the end of the experiment, the TBEA-H group showed the least reduced body weight among all the treatment groups compared to the control group (Figure 4D). DAIs climbed in all the treatment groups from day 1 to day 7, in which the DSS group climbed at the fastest speed while the TBEA-H group climbed at the slowest speed (Figure 4E).

**Figure 5:**
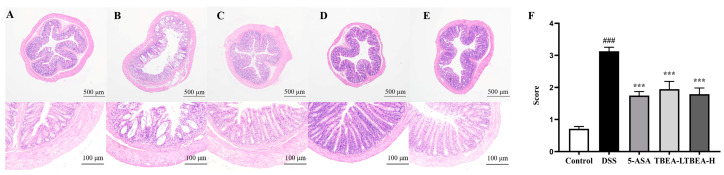
Representative images and pathology scores of HE staining in colonic tissue. HE staining results for the (**A**) control group, (**B**) model group; (**C**) 5-ASA group; (**D**) TBEA-L group; and (**E**) TBEA-H group. (**F**) Pathology score for the HE staining of the above groups. TBEA-L, TBEA-low dose (50 mg/kg); TBEA-H, TBEA-high dose (100 mg/kg); 5-ASA, 5-aminosalicylic acid (200 mg/kg). ^###^ *p* < 0.001 compared with the control group, *** *p* < 0.001 compared with the DSS group (*n* = 8).

UC inflicted severe damage to the colon, with inflammatory mucosa infiltration and massive loss of crypt and cup cells. Figure 5A shows the colon of the control group mice with neatly arranged crypt fossa, intact cup cells, and no inflammatory infiltration. However, Figure 5B shows the opposite phenomenon in the DSS group mice. After TBEA treatment, both the high-dose group and the low-dose group showed a significant reduction in the damage caused by DSS inflammation (*p* < 0.001) (Figure 5F). The above results suggest that TBEA may alleviate UC by ameliorating intestinal damage.

### 2.4. TBEA Reduces the Level of Oxidative Stress in the Colonic Tissue of UC Mice

**Figure 6:**
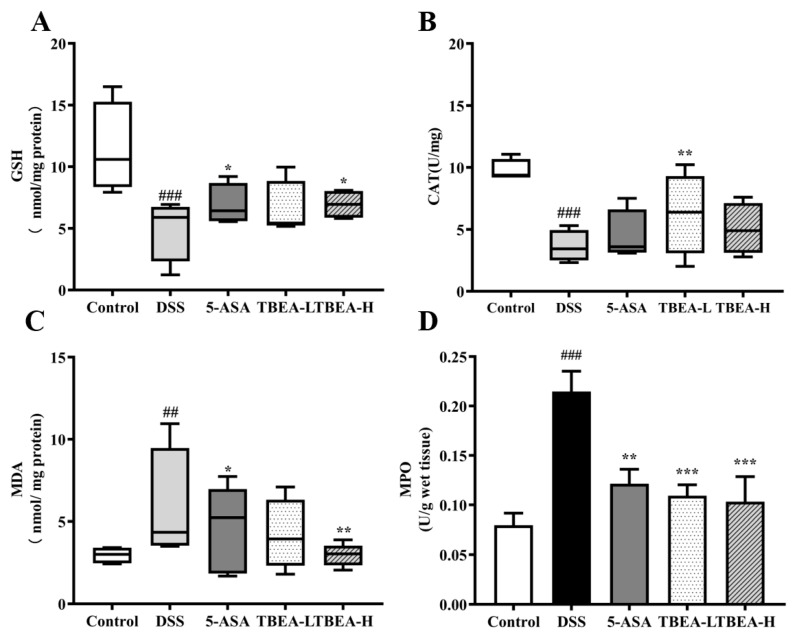
Determination of oxidative stress-related index contents: (**A**) GSH content, (**B**) CAT content, (**C**) MDA content, and (**D**) MPO activity. TBEA-L, TBEA-low dose (50 mg/kg); TBEA-H, TBEA-high dose (100 mg/kg); 5-ASA, 5-aminosalicylic acid (200 mg/kg). ^##^ *p* < 0.01, ^###^ *p* < 0.001 compared with the control group, and * *p* < 0.05, ** *p* < 0.01, *** *p* < 0.001 compared with the model group (*n* = 3).

Tissue damage is closely related to oxidative stress, and excessive free radicals in the body can damage tissue integrity. To further verify the alleviating effect of TBEA on intestinal injury in UC mice and the relationship with oxidative stress, we measured the levels of indicators related to oxidative stress in the colonic tissues of each group. Compared with the control group, DSS resulted in increased levels of malondialdehyde (MDA) and myeloperoxidase (MPO) (*p* < 0.01) and significantly decreased levels of GSH and catalase (CAT) (*p* < 0.001). Colonic glutathione (GSH) and CAT levels increased in TBEA-treated mice (*p* < 0.05), with CAT levels in the TBEA-L group close to those in the control group (Figure 6A,B). The MDA levels in the TBEA-H group were the same as those in the control group (Figure 6C). The MPO levels in the TBEA-H treatment group were almost halved compared with the DSS group (Figure 6D). It was suggested that TBEA significantly improved the level of oxidative stress in UC mice, which in turn had a protective effect on the intestine.

### 2.5. TBEA Reduces Pro-Inflammatory Cytokine Contents in the Colonic Tissue of UC Mice

**Figure 7:**
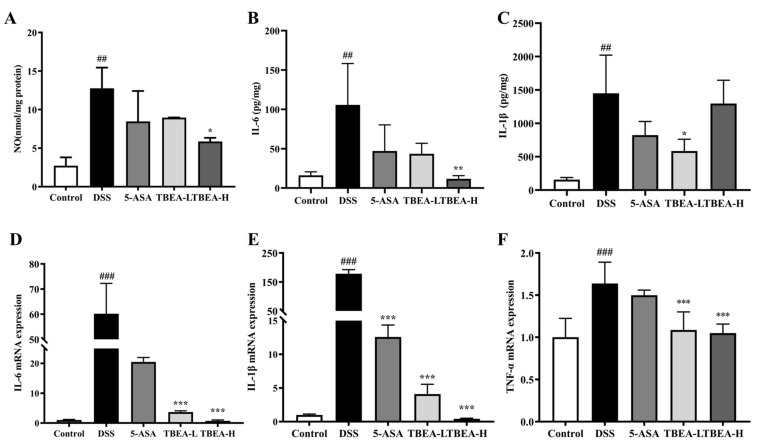
Measurement of pro-inflammatory cytokine contents: (**A**) NO content, (**B**) IL-6 content, (**C**) IL-1β content, (**D**) relative mRNA expression of IL-6, (**E**) relative mRNA expression of IL-1β, and (**F**) relative mRNA expression of TNF-α. TBEA-L, TBEA-low dose (50 mg/kg); TBEA-H, TBEA-high dose (100 mg/kg); 5-ASA, 5-aminosalicylic acid (200 mg/kg). ^##^ *p* < 0.01, ^###^ *p* < 0.001 compared with the control group, and * *p* < 0.05, ** *p* < 0.01, *** *p* < 0.001 compared with the model group (*n* = 3).

Oxidative stress and inflammation can interact with each other, and we measured the levels of pro-inflammatory cytokines in mouse colonic tissues. In the DSS group, we observed a dramatic increase in NO content and IL-6 and IL-1β expressions in the colonic tissues of UC mice compared with the control group (*p* < 0.01) (Figure 7A–C). The IL-6 content in the TBEA-H group was lower than that in the control group (Figure 7B), while the IL-1β content in the TBEA-L group decreased by more than half compared to that in the DSS group (Figure 7C). The mRNA expression levels of IL-1β, IL-6, and TNF-α in the colonic tissue were significantly increased in the DSS groups compared with their control groups (*p* < 0.001) and were effectively reduced with TBEA treatment (Figure 7D–F). The above results confirm the excellent inhibitory effect of TBEA on the inflammatory response in UC mice in vivo.

### 2.6. TBFE Inhibits the IL-6/JAK2/STAT3 Inflammatory Signaling Pathway

**Figure 8:**
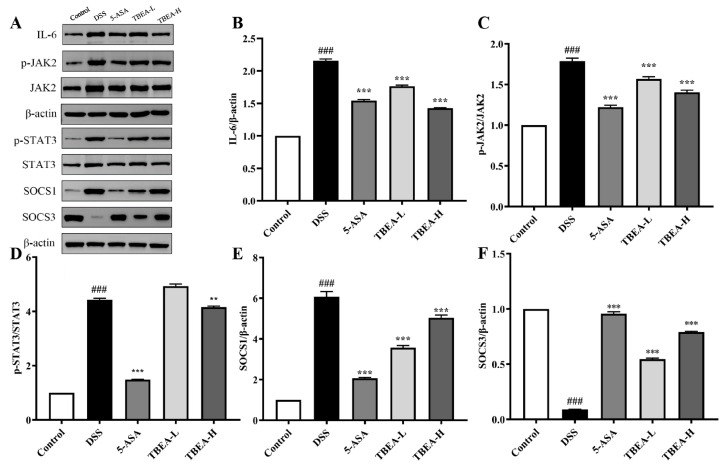
Effects of TBEA on the IL-6/JAK2/STAT3 pathways and SOCS1 and SOCS3 inflammasome expression in DSS-induced UC mice. (**A**) Expression of IL-6, p-JAK2, JAK2, p-STAT3, STAT3, SOCS1, and SOCS3 proteins in colonic tissues was determined using a Western blot. The protein expression levels of (**B**) IL-6, (**C**) p-JAK2, (**D**) p-STAT3, (**E**) SOCS1, and (**F**) SOCS3 were quantitated using ImageJ software. ^###^ *p* < 0.001 compared with the control group, and ** *p* < 0.01, *** *p* < 0.001 compared with the DSS group (*n* = 3).

Some pathways may regulate the alteration of inflammatory factors, and we examined the expressions of proteins related to the IL-6/JAK2/STAT3 inflammatory signaling pathway using western blotting. As shown in Figure 8A–D, DSS induced a significant increase in the expression of IL-6, p-JAK2/JAK2, and p-STAT3/STAT3 proteins in the colon of UC mice compared with the control group (*p* < 0.001). The expression of SOCS1 protein was reduced to half of that in the DSS group after the low-dose TBEA (50 mg/kg) treatment (Figure 8E), while the content of SOCS3 treated with a high dose of TBEA (100 mg/kg) could reverse the decrease in protein content caused by DSS to almost the control level (Figure 8F). This pathway may be one of the key pathways involved in anti-UC mechanisms caused by TBEA.

### 2.7. Influences of TBEA Treatment on Intestinal Flora

**Figure 9:**
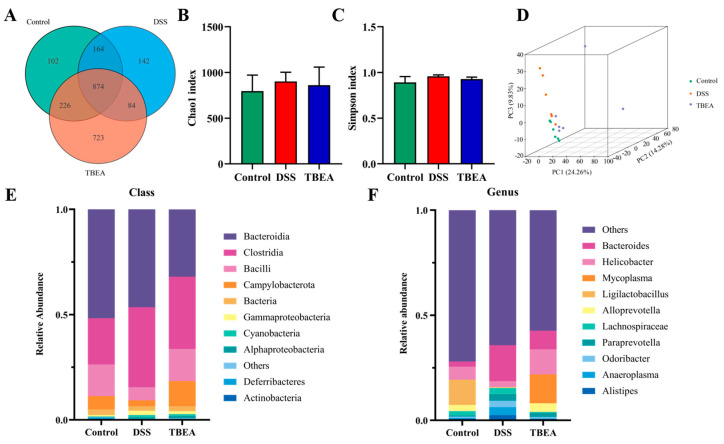
TBEA regulates the gut microbiota in DSS-induced UC mice. (**A**) Venn diagram of species in the three groups. (**B**) The Chao1 index is higher, and the species abundance is more elevated. (**C**) The Simpson index is closer to 1, and the species diversity is greater. (**D**) PCA was used to determine the β-diversity of the samples. (**E**) Relative abundances of microbiota constituents at the class level. (**F**) Relative abundances of microbiota constituents at the genus level (*n* = 6).

We used 16S rDNA high-throughput sequencing to detect rat intestinal flora. The VEEN plot (Figure 9A) shows that a total of 2315 operational taxonomic units (OTUs) were identified in the three groups, implying a strong core microbiota in this experimental mouse. Compared with the control group, the total number of OTUs decreased in the DSS group, and the number of OTUs increased after TBEA treatment. The number of OTUs specific to each group was 102 in the control group, 142 in the DSS group, and 723 in the TBEA group. It can be seen that the number of unique OTUs of mouse intestinal microorganisms was increased after TBEA treatment, which effectively prevented the alteration of mouse intestinal flora. The largest number of identical OTUs was 226 in the TBEA and control group among the three, suggesting that TBEA increased the similarity between the intestinal flora of mice with enteritis and normal mice.

α-Diversity indices are often used to characterize the richness and homogeneity of sample species to fully assess microbial communities’ diversity. The Chao1 index and Simpson index represent the abundance and diversity of intestinal flora, respectively. Figure 9B shows that TBEA treatment had no significant effect on the abundance of intestinal flora in UC mice. At the same time, the Simpson index for the three groups showed no significant differences (Figure 9C). However, the DSS group’s mean value for both the Chao1 index and the Simpson index was higher than those for the control and TBEA groups.

β-Diversity is used to reflect the differences in microbiota composition between samples. This study used principal component analysis (PCA) to analyze the databases in descending order. If the samples had a similar functional composition, then they were closer in the descending plot. The results showed that the intestinal flora composition of mice in the control and DSS groups were extremely different. Still, the composition of the TBEA group was close to that of the control group (Figure 9D), which indicated that TBEA could slow down intestinal flora disorder in UC mice.

Figure 9E shows the distribution of the top 10 groups of bacteria at the class level in terms of richness in the different groups of mice. Bacteroides and Clostridia were the dominant taxa at the class level in all groups. Compared with the control group, DSS-treated mice had an increased abundance of Clostridia and decreased abundance of Bacilli in the intestine. TBEA moderated the above changes. Figure 9F shows the distribution of the top 10 genera in terms of abundance for the different groups of mice, where harmful bacteria such as *Paraprevotella* increased after DSS induction. In contrast, the beneficial bacteria *Helicobacter* and *Ligilactobacillus* decreased. TBEA treatment reduced the abundance of the above-mentioned harmful bacteria while increasing the abundance of beneficial bacteria.

**Figure 10:**
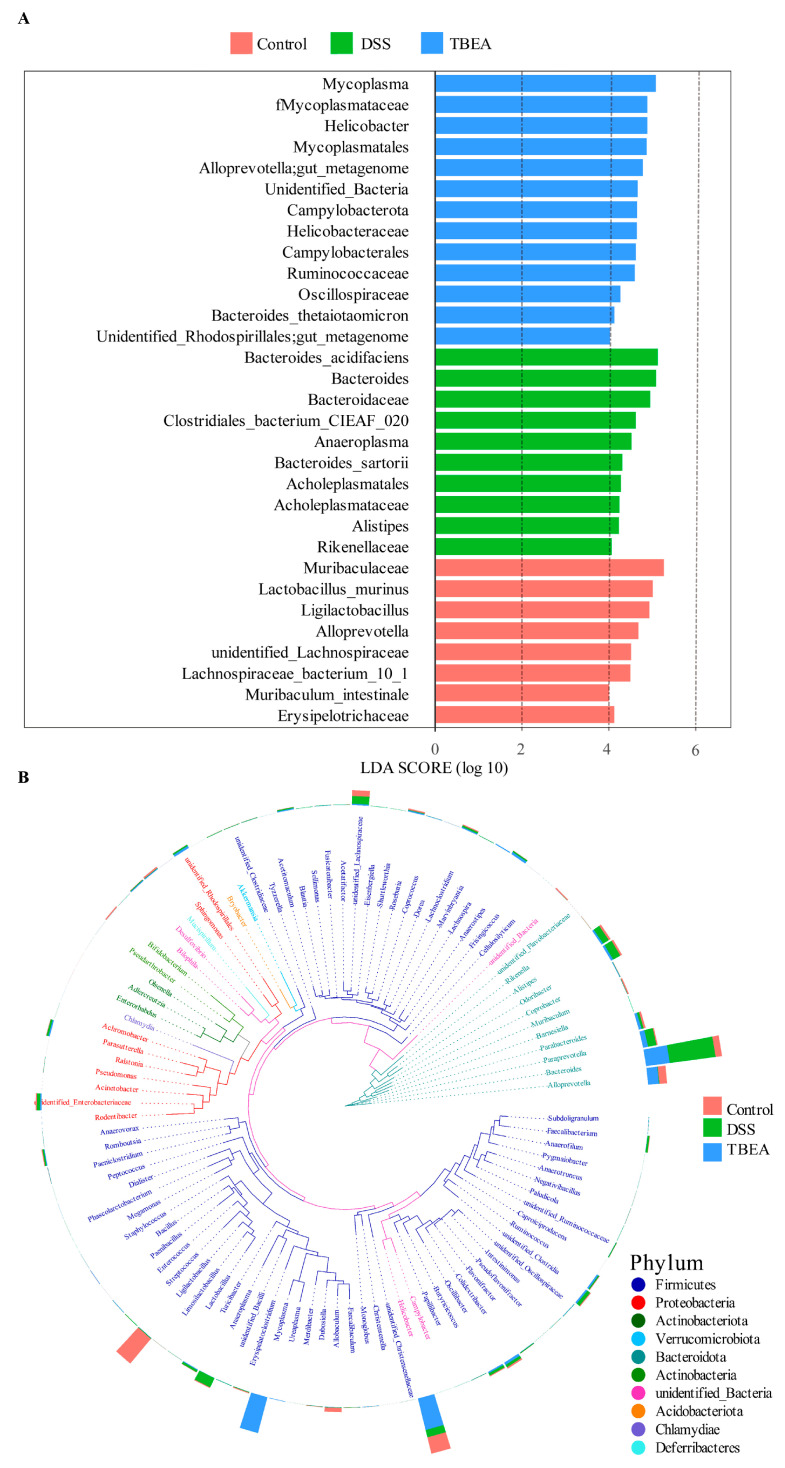
Effect of TBEA on the abundance of intestinal microbiota at the phylum level in DSS-induced UC mice. (**A**) Linear discriminant analysis (LDA). (**B**) Phylogenetic relationships of species at the phylum level based on OTU (*n* = 6).

To identify bacterial taxa that differed significantly between groups, the linear discriminant analysis effect size (LEfSe) method was used to detect the intestinal flora of mice. As shown in Figure 10A, there were 31 distinctly different species in the three groups. Combined with the horizontal clade of Top100 genera in Figure 10B, we observed that the DSS-induced changes in the gut microbiota of UC mice were mainly concentrated in *Firmicutes* and *Bacteroidota*. In contrast, TBEA treatment changed the composition and population structure of the gut flora.

## 3. Discussion

Based on the available clinical and experimental evidence, it was determined that intestinal homeostasis and inflammatory responses exacerbate each other, affecting intestinal barrier function and promoting intestinal disorders and inflammatory responses [15]. The intestinal flora is critical in intestinal homeostasis and the inflammatory response, mainly associated with inflammatory factors and oxidative stress [16]. The current clinical treatment for UC mostly uses 5-ASA, thiopurines, integrin-targeted biologics, and Janus kinase inhibitors to provide relief. However, patients are prone to drug intolerance, resulting in 15% of patients needing surgical treatment [17]. The pursuit of treatment drugs that are safe and effective with fewer side effects is essential.

TBF is a widely distributed ethnomedicine that can be used to treat various diseases. It was observed that TBF can affect ulcers caused by gastric mucosal damage by promoting mucosal recovery and reducing inflammatory cells [18], proving its anti-ulcer activity. Pandey et al. suggested that TBEA at a dose of 100 mg/kg inhibited up to 72% of castor oil-induced diarrhea in rats within four hours [19]. In addition, studies have stated that TBF can alleviate liver injury and hepatic necrosis caused by oxidative stress and reduce dysfunction, tissue damage, and arthritis produced by inflammatory factor aggregation [20]. All the above-described pharmacological effects of TBF are consistent with the possible mechanisms we proposed, confirming the potential of TBF and TBEA to treat UC.

During the characterization of TBEA, we identified some active substances. Gallic acid was one of the most distinct peaks in the TBEA qualitative profile. Gallic acid (GA) has been shown to have anti-inflammatory activity by inhibiting LPS-induced production of NO, prostaglandin E2, TNF-α, and IL-6 in macrophages in vitro [21,22]. A study confirmed that GA was capable of reversing the pathological process in acute inflammation in vivo experiments [23]. Yu et al. found that GA inhibits DSS-induced inflammatory response in ulcerative colitis by acting on the inflammasome NLRP3 [24], and they also determined that TBEA contains chebulagic acid and chebulinic acid, both of which are known to have beneficial effects in relieving inflammatory response, according to previous studies [25,26]. Therefore, we speculate that these compounds may be the main active components of TBEA that exert anti-UC effects. However, identifying which active ingredient has the best anti-UC effect needs further analysis.

*Terminalia chebula* extract can treat colitis in mice by alleviating physiological indicators such as diarrhea, bloody stools, weight loss, and colon damage [27], consistent with our study’s findings that TBEA alleviates the physiological indicators mentioned above. It is supposed that *Terminalia chebula* and *Terminalia bellirica* have similar pharmacological activities because they come from the same genus and contain similar chemical compositions. In addition, we found that colon weight tended to decrease after DSS induction. At the same time, the 5-ASA- and TBEA-H-treatment groups showed a significant recovery in colon weight that exceeded the control group’s level. This interesting phenomenon is similar to the findings of Cao [28], Ma [29], and Fay, C.N. [30] et al. However, the reason for this phenomenon is unknown and thus requires further research.

The inflammatory response stimulates the release of large amounts of reactive oxygen species (ROS) from inflammatory cells, neutrophils, and macrophages, leading to oxidative stress. The increase in ROS, in turn, stimulates the production of pro-inflammatory cytokines. Therefore, oxidative stress is a marker for detecting UC [31]. Based on our experimental results, TBEA may alleviate UC by improving intestinal oxidative stress and reducing the inflammatory response. Egyptian scholars found that hydroxytyrosol can relieve UC by enhancing oxidative stress and reducing inflammatory factors [32]. In addition, *Panax quinquefolius* polysaccharide and the ethanol extract of *Piper wallichii* also treat UC mainly by reducing inflammatory response [33,34]. 

In UC patients, the body contains pro-inflammatory and anti-inflammatory cytokines, which maintain the body’s homeostasis and influence immune cell activation and the integrity of tissue structure [35,36]. As pro-inflammatory cytokines, IL-6 and IL-1β play an important role in promoting the development of UC, with significantly elevated levels of both in UC patients [37]. TNF-α is a hallmark of DSS-induced UC and can upregulate IL-6 and IL-1β expression [38]. Jiang et al. demonstrated that oyster polysaccharides inhibited TNF-α activity from attenuating the inflammatory response and alleviating UC [39]. In addition, peanut skin procyanidins extract and peanut skin procyanidins were proven to inhibit the colon’s inflammatory response and oxidative stress by downregulating TNF-α [40]. This is sufficient to suggest the importance of this inflammatory cytokine. Our study showed the same results that TBEA was able to reduce the severity of UC disease by inhibiting the production of TNF-α. 

Interleukin-6 (IL-6) is an important pro-inflammatory cytokine. It has been demonstrated that there is an increase in the level of IL-6 when the body suffers from UC [41], which is consistent with our study. After measuring the content of IL-6 in the colonic tissues from TBEA-treated UC mice, we observed that TBEA significantly inhibited its production. Moreover, Lactobacilli, which became more abundant after TBEA treatment in this experiment, was proved to affect the secretion of IL-6 production [42]. Therefore, we believe that IL-6 is the key cytokine in alleviating UC with TBEA and suggest analyzing its downstream cytokines to investigate the anti-UC mechanism of TBEA treatment. The JAK/STAT pathway, which is one of the critical signaling pathways in autoimmune diseases, can act on a variety of cytokines [43,44]. STAT-3 is phosphorylated as a substrate of JAK2, regulating gene transcription and expression of inflammatory cytokines and promoting further inflammation [45]. The suppressor of cytokine signaling-1 (SOCS 1) is a key inhibitor of cytokine signaling, and its expression is regulated by phosphorylated STAT3 [46]. SOCS3, in turn, affects this pathway by inhibiting JAK2 activity [47]. Clinical trials have demonstrated that enhancing the intestinal barrier function by affecting the IL-6/STAT3 signaling pathway can attenuate UC-induced intestinal injury [48]. Clinical trials using JAK inhibitors as therapeutic agents for IBD and UC are also unfolding. For example, research on the PAN-JAK inhibitor TD-1473, which can exert anti-UC effects by regulating JAK2, is in phase III of the clinical study [49]. Various TCMs, such as Gegen Qinlian decoction [50] and *Chrysanthemum morifolium* polysaccharide [51], have been suggested to alleviate UC through this pathway. Our study results showed that TBEA inhibited the activation of JAK2 and STAT3 while increasing the level of SOCS3 in colonic tissues, further inhibiting the signaling of this pathway. The above suggests that the anti-UC activity of TBEA may be attributed to its ability to inhibit this signaling pathway in an inflammatory environment. 

The intestinal microbiome is a key determinant of intestinal health and is directly associated with the integrity of the intestinal epithelial barrier layer [52]. We assessed the effect of TBEA administration on the gut microbiome. At the class and genus level, we observed an increased abundance of UC-associated Actinomycetes and Bacteroidetes species and a relative decrease in Lactobacillus. TBEA treatment increased the abundance of Lactobacillus in the intestine. Lactobacilli are often considered beneficial probiotic thick-walled phylum microorganisms whose products inhibit the production of inflammatory factors, including IL-Ιβ, IL-6, and TNF-α. Lactobacilli may also promote beneficial shifts in the composition of the intestinal flora, producing antibiotic compounds that may prevent the colonization of the intestine by potentially pathogenic bacteria [53]. The above observations suggest that TBEA may stabilize intestinal homeostasis by reducing the abundance of harmful flora in the intestine and suppressing inflammation by beneficial bacterial species products. It provides a basis for TBEA to treat UC by regulating intestinal flora. 

## 4. Materials and Methods

### 4.1. Chemicals

TBF (Sample number:20211216) was obtained from Yunnan Province, China. It was authenticated by Dr. Gao Zhou at the Hubei University of Technology and then stored in a dry 4 °C environment. DSS was produced by MP Biomedicals (molecular weight: 36,000–50,000, Solon, OH, USA), and 5-aminosalicylic acid(5-ASA) was provided by Shanghai Yien Chemical Technology Co., Ltd. (Shanghai, China). The occult blood test kit, MDA, CAT, GSH, MPO, and nitric oxide assay kit were purchased from the Beyotime Institute of Biotechnology. Enzyme-linked immune sorbent assay (ELISA) kits for IL-6 and IL-1β, were produced by Beijing Solarbio Science & Technology Co., Ltd. (Beijing, China). ChamQ Universal SYBR qPCR Master Mix (Q711-02) and HiScript III RT SuperMix for qPCR (+gDNA wiper) (R323-01) were bought from Vazyme Biotech Co., Ltd. (Nanjing China). Anti-p-STAT3 (AB267373) and AntiSOCS1 (AB280886) were from Abcam Co., Ltd. (Shanghai, China). Anti-IL-6 (GB11117), Anti-JAK2 (GB11325), Anti-p-JAK2 (GB114585), Anti-SOCS3 (GB113792), Anti-STAT3 (GB11176), and Anti-ACTIN (GB15001) were from Servicebio Co., Ltd. (Wuhan, China). Horseradish peroxidase-conjugated anti-rabbit IgG was purchased from Servicebio Biotechnology (Wuhan, China). The other analytical grade solvents including 95% ethanol were supplied by Sinopharm Chemical Reagent Co. Ltd. (Shanghai, China).

### 4.2. Preparation and Evaluation of the TBF Extracts

Dried TBF was crushed using a grinder and then sieved through a 200 mesh. Then, the TBF power was extracted using ultrasonication with 95% ethanol three times for 30 min each. The supernatant obtained after filtration was evaporated with a rotary evaporator to obtain TBFE. TBFE was dissolved in deionized water and extracted successively with petroleum ether, dichloromethane, ethyl acetate, and *n*-butanol to obtain various fractions, including the aqueous phase.

### 4.3. High-Performance Liquid Chromatography Analysis

An HPLC analysis was performed on the various TBF fractions. TBFE and five extract fractions were diluted with 1 mg/mL of methanol. The samples were filtered through a 0.22 μm microporous membrane and injected into a 5C18-MS-II (5 μm particle size, 4.6 × 250 mm, COSMOSIL) HPLC column. The mobile phase was 0.1% formic acid aqueous solution (*v*/*v*) as eluent A and acetonitrile as eluent B. The gradient program was 0~5 min, 5% B; 5~15 min, 5~15% B; 15~20 min 15% B; 20~ 35 min, 15~20% B; 35~45 min, 20~60% B; 45~50 min, 60~5% B; and 50~55min, 5% B. The mixes for the preparation of the standards for chebulic acid, gallic acid, corilagin, chebulagic acid, ellagic acid, and chebulinic acid were 1 mg/mL, 2 mg/mL, 1 mg/mL, 2 mg/mL, 1 mg/mL, and 2.5 mg/mL, respectively. Absorption of 100 μL, 100 μL, 100 μL, 200 μL, 100 μL, and 200 μL were mixed in the above sequence, respectively, and characterized under the same chromatographic conditions as the experimental samples.

### 4.4. Cell Viability Determination

Mouse mononuclear macrophages RAW264.7 (No. GDC0143) were obtained from the China Center for Type Culture Collection (Wuhan University, Wuhan, China) in RPMI 1640 medium containing 15% fetal bovine serum (FBS) and 1% penicillin–streptomycin solution and placed at 37 °C in a 5% CO_2_ incubator.

Cell viability was determined using an MTT assay. Logarithmic growth phase cells were prepared as cell suspensions, inoculated into 96-well plates at a density of 1 × 10^4^/mL, and divided into blank, control, and experimental groups, where the blank group had no cells. After 24 h of incubation, the supernatant was aspirated, and 100 μL of TBFE and five extract fractions solutions at concentrations of 0–200 μg/mL were given to the experimental and blank groups. At the same time, the corresponding volume of culture medium was added to the control group. After 24 h of incubation, MTT at a final concentration of 1 mg/mL was added to the experimental and control groups, and no MTT was added to the blank group. The supernatant was discarded after 4 h of incubation, and then 100 μL of DMSO was added to all groups. The reaction was performed for 30 min under light-proof conditions, and the absorbance was measured at 570 nm using a microplate reader. The cell survival rate was calculated as Cell survival rate = (OD experimental group-OD blank group/OD control group-OD blank group) × 100%.

### 4.5. NO Content Determination

Cells were inoculated in 96-well plates with RAW264.7 at a density of 5 × 10^5^. The cells were divided into control, LPS, 5-aminosalicylic acid (5-ASA), and extract experimental groups. After 24 h of normal incubation, the supernatant was discarded. Drugs at 5–40 μg/mL concentrations were added to each group, and the control and LPS groups were added with culture solution. After incubation for one hour, LPS with a final concentration of 1 μg/mL was added to all subjects except the control group. Greiss reagent was added and kept for 20 min with protection from light after 24 h incubation. The absorbance was measured at 540 nm using microplate reader standardization, and the standard curve of sodium nitrite determined the concentration of NO in the supernatant of each group.

### 4.6. Animals and Experimental Design

Forty male C57BL/6 mice (6–8 weeks old, 20 ± 5 g) were purchased from the Hubei Provincial Center for Disease Control and Prevention. All mice were placed in a constant temperature environment at 25 °C and fed water and standard chow for 3 d to acclimatize to the environment. To determine the optimal dose to be administered in animal experiments, the equivalent human amount (3–9 g) at the time of administration was calculated by referring to the Chinese Pharmacopoeia, which was converted to the applicable dose in mice according to the human–mouse drug dose conversion principle [10]. The final amounts of TBEA for mice were determined to be 50 mg/kg and 100 mg/kg, respectively, of which 100 mg/kg was converted from the human dose of 6 g of TBF. The mice were weighed and recorded before the beginning of the experiment and randomly divided into a control group, model group, 5-ASA group (200 mg/kg), TBEA low-dose group, and TBEA high-dose group, with eight mice in each group. The mice in the control group were given water, while the other group was given 2.5% DSS solution instead of water for 6 d. From day 1 to day 6, 5-ASA and two doses of TBEA were treated with intragastric gavage in the positive control and TBEA groups, respectively. On day 7, DSS was replaced with drinking water in the model group, while the treatment with 5-ASA in the positive control group and TBEA in the TBEA groups was continued. On day 8 of the experiment, the mice were sacrificed using CO_2_, and the colon samples were rapidly obtained after saline rinsing and measuring the length and weight. A 1 cm colon length was fixed in 4% paraformaldehyde, while the other portion was stored at −80 °C for further analysis. All animal experiments were supervised and approved by the Animal Care and Use Committee at Hubei University of Technology (HBUTLL20230033, Wuhan, China).

### 4.7. DAI

During the experiment, the body weight and fecal consistency were recorded daily for each group of mice, and the feces were collected and measured using the o-toluidine method to detect the occult blood. The mean values for the three indexes were calculated according to the relevant criteria [54,55,56] listed in Table 1 to obtain the final DAI.

### 4.8. Histological Analysis of the Colon

Distal colon specimens were fixed in 4% paraformaldehyde for 48 h, dehydrated in an ethanol gradient, paraffin-embedded, sectioned to 5 μm, and stained with hematoxylin and eosin (H&E). Three different experimenters performed the histological scoring, according to Table 2, which was summarized from relevant references [57,58,59].

### 4.9. Determination of Oxidative Stress-Related Indicators in the Mice Colon

The colon tissue from each group of mice was randomly selected and homogenized with 0.9% saline at 10,000× *g*; the supernatant was obtained after centrifugation for 10 min. The protein concentration was determined using the BCA protein concentration assay kit. GSH, CAT, MDA, and MPO contents were measured according to the kit’s instructions.

### 4.10. Measurement of Colonic Inflammatory Factor Levels in Mice

The contents of IL-6 and IL-1β in colonic tissues were determined using an enzyme-linked immunosorbent assay (ELISA). The colon tissues from mice in each group were randomly obtained and homogenized with 0.9% saline, and the supernatant was obtained after centrifugation at 5000× *g* for 10 min. According to the instructions for the IL-1β and IL-6 ELISA kits, the content of inflammatory factors in the colonic tissue from each group of mice was measured. The NO content in the tissues was also determined according to the instructions provided for the NO kit.

### 4.11. Analysis of Inflammatory Factors using qPCR

The total RNA from each group of mice was extracted using TRIzol. The purity and concentration of the extracted RNA were determined using NanoDrop2000 (Thermo Fisher Scientific, Waltham, MA, USA). The cDNA samples were synthesized with RNA reverse transcription using the HiFiScript cDNA Synthesis Kit. UltraSYBR Mixture was prepared for real-time PCR reaction system for quantitative analysis. The reaction mixture was made by mixing 0.2 μL of upstream primer (10 μM), 0.2 μL of downstream primer (10 μM), 1 μL of the diluted sample, 3.6 μL of ddH2O, and 5 μL of UltraSYBR mixture (2×). The primers used for qRT-PCR are listed in Table 3, and these primers were synthesized by Tsingke Biotechnology Co., Ltd. RNA expression levels were quantified using a real-time system (Applied Biosystems, Foster City, CA, USA). The thermal cycling conditions were set at the manufacturer’s recommendation of 40 cycles. Each gene’s cycle thresholds (Ct) were recorded and normalized with the Ct value of β-actin using the 2-^∆∆CT^ method.

### 4.12. Western Blot Analysis

A Western blot was used to detect the expression of IL-6, JAK2, p-JAK2, STAT3, p-STAT3, SOCS1, and SOCS3 proteins in colon tissues. Colon tissues from each group were homogenized, and RIPA reagent was added to lysate cells. After a 20 min wait, the supernatant was obtained using centrifugation at 12,000× *g* for 10 min. Protein quantification was performed using the BCA method. Equal amounts of proteins (20 μg per sample) were electrophoresed on 10% sodium dodecyl sulfate–polyacrylamide gel, followed by immunoblotting to transfer proteins to polyvinylidene difluoride (PVDF) membranes. The membranes were closed with 5% skim milk for two hours at room temperature and washed with TBST thrice for 20 min. The membranes were incubated with the primary antibody overnight at 4 °C, washed with TBST, and incubated with the secondary antibody to detect the protein bands using an electrochemiluminescence (ECL) system. The grayscale value for each band was determined using Image J software, and the ratio of each protein to the internal reference (β-actin) was used as the relative expression of each protein.

### 4.13. Gut Microbiota Analysis and 16S rDNA Sequencing 

Mouse feces were collected from the control, DSS, and TBEA-H groups, and the total DNA from each sample was extracted using a CTAB reagent. Then, 2% agarose gel electrophoresis was used to determine the purity and concentration of DNA, which was finally amplified at a concentration of 1 ng/μL. Bacterial 16SrDNA in the V3-V4 region was amplified with universal gene primers: 515F (5߰-GTGCCAGCMGCCGCGGTAA-3′) and 806R (5߰-GGACTACHVGGGTWTCTAAT-3߰). Sequences were clustered into operational taxonomic units (OTUs) with 97% agreement using the clustering program USEARCH (version 7.0) with double-indexed amplification and sequencing methods on the NovaSeq sequencing platform. Species annotation analysis was performed using the Mothur method with the SSUrRNA database of SILVA138.1, and the community composition of each sample was counted at the phylum and genus levels, respectively. Finally, the data for each sample were homogenized, and the Chao1 and Simpson indices were calculated using Qiime software (Version 1.9.1). R software (Version 4.1.2) was used to plot te PCA and perform inter-group variation analysis of the Beta diversity index.

### 4.14. Statistical Analysis

SPSS 22.0 software was used for one-way analysis of variance (ANOVA) followed by Fisher’s least significant difference test for multiple comparisons. Differences between groups were considered statistically significant at *p* < 0.05.

## 5. Conclusions

In this study, we investigated the alleviating effect of TBEA on UC and its mechanism of action using a DSS-induced UC mouse model. Our results showed that TBEA mainly contains chebulic acid, gallic acid, corilagin, chebulagic acid, ellagic acid, and chebulinic acid. TBEA reduced weight loss and diarrhea and improved intestinal damage in UC rats. The mechanism of action underlying TBEA’s anti-UC activities is mainly involved in antioxidant capacity enhancement, inflammatory response suppression, IL-6/JAK2/STAT3 signaling pathway inhibition, and gut microbiota regulation. In summary, TBEA can be used as a medicinal plant to alleviate UC and can be applied as a novel anti-UC drug candidate.

## Figures and Tables

**Table 1:** Criteria for scoring DAI.

Score	Rate of Weight Change	Stool Formation	Occult Blood Status
0	0%	Normal	No color development within 2 min
1	1–5%	Soften	Change from light green to green after 10 s
2	5–10%	Loose	Change from light green to blue–brown
3	10–15%	Unshaped	Change from blue–brown to black–brown
4	>15%	Diarrhea	Immediately turns blue–black–brown

**Table 2:** Histological scoring.

Score	Inflammatory Factor Infiltration	Epithelial Cell Integrity
0	Normal	Normal
1	Local inflammatory infiltration of crypts	Partial loss of cup cells
2	Inflammatory infiltration to the base of the crypt	Partial disappearance of the crypt
3	Inflammatory infiltration into the mucosa	Large absence of crypt fossa
4	Inflammatory infiltration into the submucosa	The disappearance of crypt fossa

**Table 3:** Primers used in qPCR.

Target Gene	Upstream Primer (5′-3′)	Downstream Primer (5′-3′)
IL-6	GAGTCACAGAAGGAGTGGCTAAGGA	CGCACTAGGTTTGCCGAGTAGATCT
IL-1β	TGCCACCTTTTGACAGTGATG	CATCTCGGAGCCTGTAGTGC
TNF-α	GCATGGTGGTGGTTGTTTCTGACGAT	GCTTCTGTTGGACACCTGGAGACA
β-actin	GCAGGAGTACGATGAGTCCG	ACGCAGCTCAGTAACAGTCC

## Data Availability

The data used in this study were submitted to the NCBI, and the Bioproject number is PRJNA990353.

## Sample Availability

Samples of the compounds are available from the authors.
